# Porphyrins as Corrosion Inhibitors for N80 Steel in 3.5% NaCl Solution: Electrochemical, Quantum Chemical, QSAR and Monte Carlo Simulations Studies

**DOI:** 10.3390/molecules200815122

**Published:** 2015-08-18

**Authors:** Ambrish Singh, Yuanhua Lin, Mumtaz A. Quraishi, Lukman O. Olasunkanmi, Omolola E. Fayemi, Yesudass Sasikumar, Baskar Ramaganthan, Indra Bahadur, Ime B. Obot, Abolanle S. Adekunle, Mwadham M. Kabanda, Eno E. Ebenso

**Affiliations:** 1 State Key Laboratory of Oil and Gas Reservoir Geology and Exploitation, Southwest Petroleum University, Chengdu 610500, Sichuan, China; E-Mails: vishisingh4uall@gmail.com (A.S.); yhlin28@163.com (Y.L.); 2 Department of Chemistry, LFTS, Lovely Professional University, Phagwara, Punjab 144402, India; 3 Department of Chemistry, Indian Institute of Technology (Banaras Hindu University), Varanasi 221005, India; E-Mail: maquraishi.apc@itbhu.ac.in; 4 Department of Chemistry, Faculty of Science, Obafemi Awolowo University, Ile-Ife 220005, Nigeria; E-Mails: waleolasunkanmi@gmail.com (L.O.O.); sadekpreto@gmail.com (A.S.A.); 5 Department of Chemistry, School of Mathematical & Physical Sciences, North-West University (Mafikeng Campus), Private Bag X2046, Mmabatho 2735, South Africa; E-Mails: fomololaesther@yahoo.com (O.E.F.); sasikumar.phd@gmail.com (Y.S.); ramaganthanbaskar@gmail.com (B.R.); bahadur.indra@gmail.com (I.B.); Mwadham.Kabanda@nwu.ac.za (M.M.K.); 6 Material Science Innovation & Modelling (MaSIM) Research Focus Area, Faculty of Agriculture, Science and Technology, North-West University (Mafikeng Campus), Private Bag X2046, Mmabatho 2735, South Africa; 7 Center of Research Excellence in Corrosion, King Fahd University of Petroleum and Minerals, Dhahran 31261, Saudi Arabia; E-Mail: proffoime@yahoo.com

**Keywords:** N80 steel, EIS, polarization, porphyrins, SECM, SEM, QSAR, Monte Carlo simulations, quantum chemical studies

## Abstract

The inhibition of the corrosion of N80 steel in 3.5 wt. % NaCl solution saturated with CO_2_ by four porphyrins, namely 5,10,15,20-tetrakis(4-hydroxyphenyl)-21*H*,23*H*-porphyrin (HPTB), 5,10,15,20-tetra(4-pyridyl)-21*H*,23*H*-porphyrin (T4PP), 4,4′,4″,4‴-(porphyrin-5,10,15,20-tetrayl)tetrakis(benzoic acid) (THP) and 5,10,15,20-tetraphenyl-21*H*,23*H*-porphyrin (TPP) was studied using electrochemical impedance spectroscopy (EIS), potentiodynamic polarization, scanning electrochemical microscopy (SECM) and scanning electron microscopy (SEM) techniques. The results showed that the inhibition efficiency, η% increases with increasing concentration of the inhibitors. The EIS results revealed that the N80 steel surface with adsorbed porphyrins exhibited non-ideal capacitive behaviour with reduced charge transfer activity. Potentiodynamic polarization measurements indicated that the studied porphyrins acted as mixed type inhibitors. The SECM results confirmed the adsorption of the porphyrins on N80 steel thereby forming a relatively insulated surface. The SEM also confirmed the formation of protective films of the porphyrins on N80 steel surface thereby protecting the surface from direct acid attack. Quantum chemical calculations, quantitative structure activity relationship (QSAR) were also carried out on the studied porphyrins and the results showed that the corrosion inhibition performances of the porphyrins could be related to their E_HOMO_, E_LUMO_, ω, and μ values. Monte Carlo simulation studies showed that THP has the highest adsorption energy, while T4PP has the least adsorption energy in agreement with the values of σ from quantum chemical calculations.

## 1. Introduction

Corrosion is a major problem in oil and gas industries and many factors have to be taken into account when attempting to solve the corrosion problems facing these industries [1]. Water and carbon dioxide produced or injected into oil wells for secondary recovery purposes can cause severe corrosion of the steels in the oil well. It has been reported that approximately 60% of oilfield failures are related to CO_2_ corrosion, mainly due to the current inadequate predictive capability for this form of corrosion and the poor resistance of carbon and low alloy steels to this type of corrosive attack [2,3,4,5]. Steel corrosion in a CO_2_ environment is more worrisome than in other media because, apart from promoting uniform corrosion, CO_2_ also promotes localized corrosion, which is even more detrimental [1]. N80 casing steel is used for H_2_S exposure situations in some oil fields around the world; furthermore, the reservoir usually contains CO_2_, which makes the corrosion situation often more complex. N80 casing steel can resist H_2_S, but does not resist CO_2_ effectively in the reservoir. This motivated us to investigate the inhibition of corrosion of N80 casing steel in CO_2_-containing environments.

Inhibitors are substances which when added in small concentrations to the aggressive solutions used in industries will reduce the rate of metal corrosion [6]. Inhibitors reduce corrosion rate usually by adsorbing on the surface of the metal to form a compact protective or passive film [7]. Organic compounds that contain N, S, and O heteroatoms, especially in conjunction with aromatic or other π-electronic systems, have been found to possess excellent anticorrosion potential [8]. The porphyrin molecule, as a Lewis acid with a network of conjugated π-electron systems and four nitrogen atoms at its core, possesses the molecular frame of a potential corrosion inhibitor. It is a tetradentate chelating agent with strong bonding capability and evident ability to interact with surfaces by several physical and/or chemical mechanisms [9]. Metal-porphyrin complexes are versatile model compounds for metalloenzymes and electron transport in biological systems. Porphyrins also find numerous applications as ligands for the spectrophotometric determination of cations, stationary phases in high-pressure liquid chromatography (HPLC), biosensors, catalysis, photovoltaic cells and membrane components for ion selective electrodes [10,11,12,13]. Their applications as corrosion inhibitors for carbon steel in aqueous mineral acid have also been reported [14,15,16]. A number of reports on the corrosion inhibition properties of different porphyrin molecules have revealed that porphyrin molecules can reconfigure the electron distribution of their conjugated aromatic rings in order to create ordered molecular layers on electrode surfaces. These molecular layers serve as the protective film, which prevents the diffusion of electroactive species towards the metal surface. The adsorption of porphyrin molecules and their corrosion inhibition properties are affected by a number of factors such as peripheral functional groups, steric hindrance and electron density at donor centers [16].

**Figure 1:**
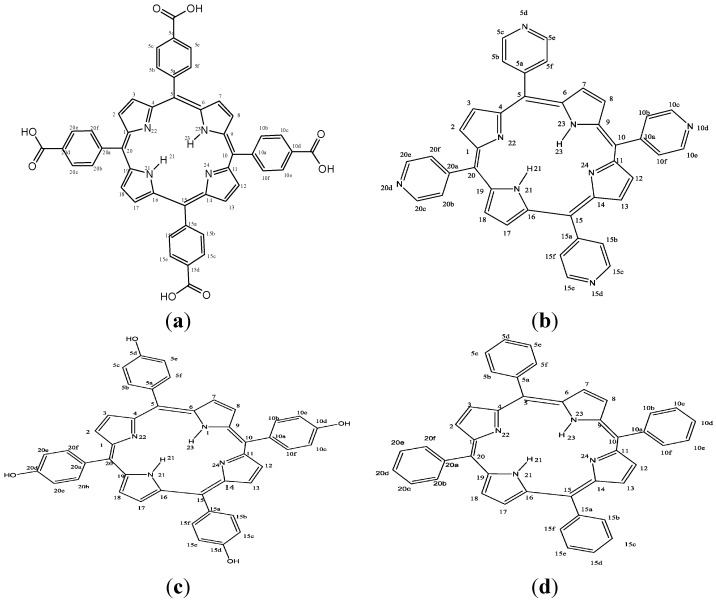
Molecular structures of the studied porphyrins. (**a**) 4,4,4,4-(porphyrin-5,10,15,20-tetrayl)-tetrakis(benzoic acid (HPTB); (**b**) 5,10,15,20-tetra(4-pyridyl)-21*H*,23*H*-porphyrin (T4PP); (**c**) 5,10,15,20-tetrakis(4-hydroxyphenyl)-21*H*,23*H*-porphyrin (THP); (**d**) 5,10,15,20-tetraphenyl-21*H*,23*H*-porphyrin (TPP).

In the present study, four porphyrin compounds namely 4,4′,4″,4‴-(porphyrin-5,10,15,20-tetrayl)tetrakis(benzoic acid) (HPTB), 5,10,15,20-tetra(4-pyridyl)-21*H*,23*H*-porphyrin (T4PP), 5,10,15,20-tetrakis(4-hydroxyphenyl)-21*H*,23*H*-porphyrin (THP) and 5,10,15,20-tetraphenyl-21*H*,23*H*-porphyrin (TPP) were investigated for their inhibition potentials on the corrosion of N80 steel in 3.5 wt. % NaCl solution saturated with CO_2_. The four compounds have the same porphyrin nucleus but different substituents at positions 5, 10, 15 and 20. Therefore, the effects of benzoic acid, pyridinyl, phenolic and phenyl substituents on the corrosion inhibition properties were examined. Experimental techniques such as electrochemical impedance spectroscopy (EIS), potentiodynamic polarization, scanning electrochemical microscopy (SECM) and scanning electron microscopy (SEM) together with theoretical methods such as quantum chemical calculations, quantitative structure activity relationship (QSAR) and Monte Carlo simulation have been used to study the corrosion inhibition mechanism and determine the inhibition efficiencies of the studied porphyrins. To the best of our knowledge, the set of porphyrin compounds used in the present work has not been considered as corrosion inhibitors in any previous study. The structures of the four porphyrins used as corrosion inhibitors in the present study are shown in Figure 1.

## 2. Results and Discussion

### 2.1. EIS Measurements

**Figure 2:**
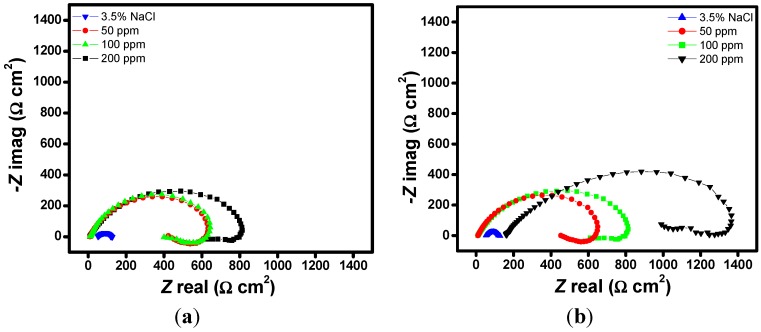

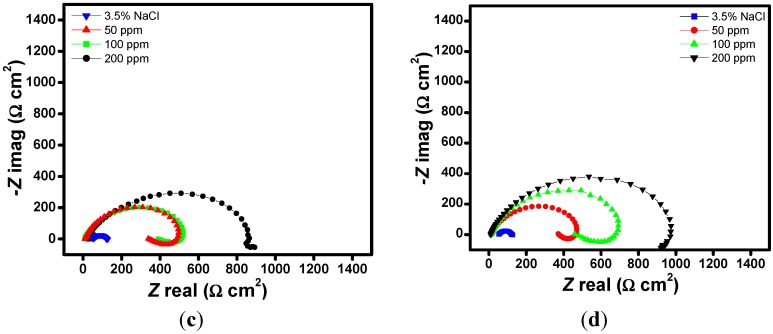
Nyquist plots of N80 steel in 3.5% NaCl saturated with CO_2_ without and with various concentrations of (**a**) HPTB; (**b**) T4PP; (**c**) THP and (**d**) TPP.

**Figure 3:**
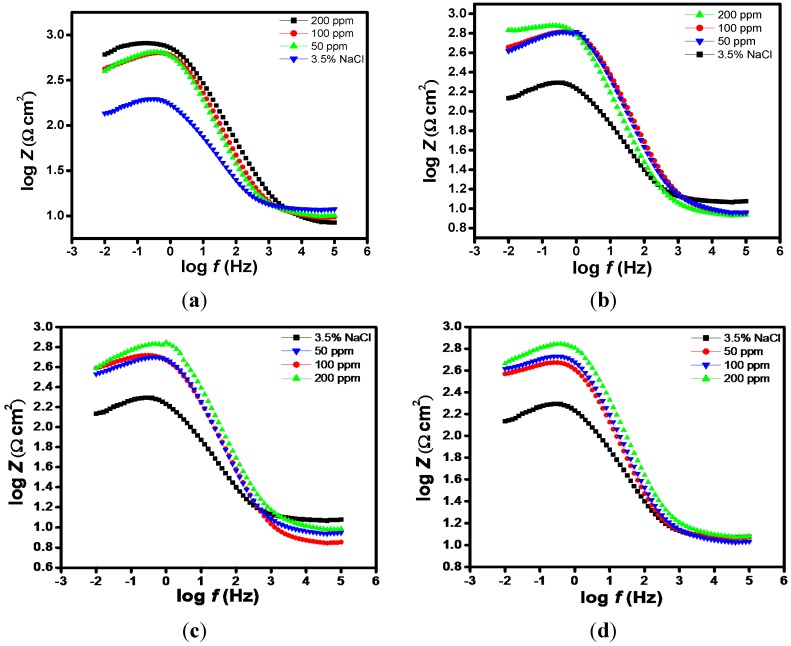
Bode modulus plots of N80 steel in 3.5% NaCl saturated with CO_2_ without and with various concentrations of (**a**) HPTB; (**b**) T4PP; (**c**) THP and (**d**) TPP.

**Figure 4:**
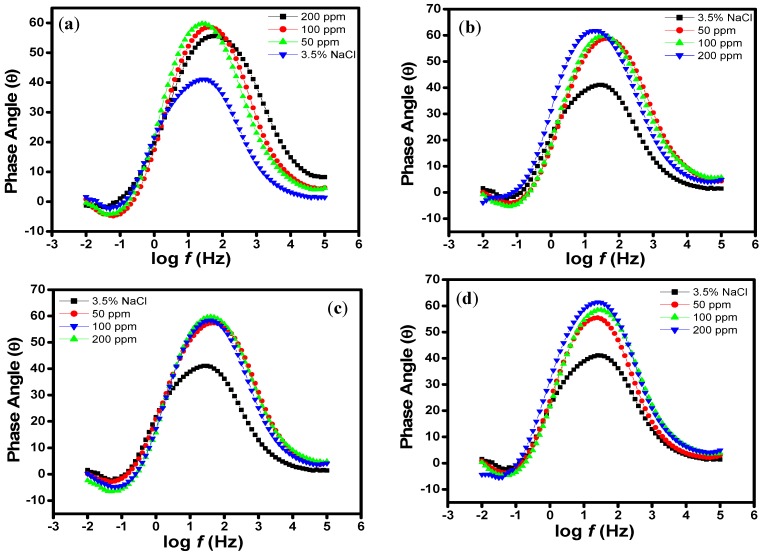
Bode phase angle plots of N80 steel in 3.5% NaCl saturated with CO_2_ without and with various concentrations of (**a**) HPTB; (**b**) T4PP; (**c**) THP and (**d**) TPP.

Impedance spectra for N80 steel in CO_2_ saturated 3.5% NaCl solution in the absence and presence of different concentrations of the studied porphyrins are shown in the form of Nyquist plots (Figure 2a–d), Bode-modulus plots (Figure 3a–d) and in the phase angle-frequency plots (Figure 4a–d). The Nyquist plots contain depressed semicircles with the center under the real axis. The plots contain one capacitive loop in the high frequency (HF) zone and one inductive loop in the lower frequency (LF) zone. Such behavior is characteristic of solid electrodes often referred to as frequency dispersion and has been attributed to roughness and other inhomogeneities of solid electrode surface [8]. The LF inductive loop may be a consequence of the layer stabilization by-products of the corrosion reaction at the electrode involving inhibitor molecules and their reactive products [8,17]. As far as the inhibition process is concerned, it is generally assumed that the adsorption of the inhibitors at the metal/solution interface is the initial step in the inhibition mechanism. Considering the dependence of inhibition efficiency on inhibitor concentration as represented in Figure 1a–d, it seems likely that the inhibitor molecules adsorb on the steel surface and block the available active sites on the surface. In other words, these inhibitor molecules decrease the number of exposed active sites that could be involved in steel dissolution.

**Figure 5:**
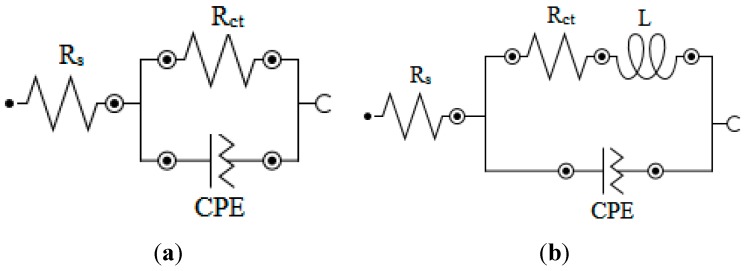
Equivalent circuits employed for the fitting of impedance spectra of N80 steel in 3.5% NaCl saturated with CO_2_ (**a**) without the inhibitors (**b**) with inhibitors.

The Nyquist and Bode plots showed typical one time constants with a single negative slope and single inflection in Bode plots. The impedance spectra for 3.5% NaCl solution was analyzed by fitting to the equivalent circuit model of the form in Figure 5a at one time constant, which has been successfully used elsewhere to describe iron/acid interface [18,19]. In this equivalent circuit, *R*_s_ is the solution resistance, *R*_ct_ is the charge transfer resistance, and CPE is a constant phase element. The Nyquist plots of the inhibitor containing systems were analyzed by another equivalent circuit model having inductance (*L*) in addition to *R*_s_, *R*_ct_ and CPE as shown in Figure 5b. The presence of *L* in the impedance spectra in the presence of investigated inhibitors indicated that the dissolution of iron occurred by direct charge transfer at the inhibitor adsorbed electrode surface. The double-layer capacitance on real cells often behave like a CPE rather than a pure capacitor due to roughness, porosity or inhomogeneity of the surface. The CPE is often used in an equivalent circuit in place of capacitance in order to ensure a more accurate curve fitting [20]. The capacitance associated with the CPE can be expressed as [21,22,23,24]:

(1) ZCPE=(YoRct1−n)1n

where, *Y_o_* is the CPE constant (in Ω^−1^·s^n^·cm^−2^), *j* is the square root of −1, and *n* is the phase shift, which can be used as a gauge of the heterogeneity or roughness of the surface. The CPE can be expressed by the values of *n*, such that for resistance (*n* = 0, *Y* = *R*), capacitance (*n* = 1, *Y* = *C*), inductance (*n* = −1, *Y* = *L*) or Warburg impedance (*n* = 0.5, *Y* = *W*). The low values of the associated chi-square indicate that the circuit was able to fit the experimental data accurately. The impedance parameters such as solution resistance (*R*_s_), charge transfer resistance (*R*_ct_), *n*, *L* and inhibition efficiency, η% are listed in Table 1. The values of η% were calculated using the equation:

(2) $$\text{η}\%=\frac{R_{\text{ct,i}}-R_{\text{ct,0}}}{R_{\text{ct,i}}}\times 100$$

where, *R*_ct,i_ and *R*_ct,0_ are charge transfer resistances in the presence and absence of the inhibitor, respectively. The values of the charge transfer resistance in the presence of the inhibitors are generally larger than that of the uninhibited system (Table 1). This implies that the corrosion rate is reduced in the presence of the inhibitors due to decrease in the area of active surface necessary for corrosion reaction [25,26]. It is clear from Table 1 that the greatest inhibition effect was observed at 200 ppm of T4PP with *R*_ct_ = 1356 Ω·cm^2^. The decreasing order of the *R*_ct_ values at 200 ppm is T4PP > TPP > THP > HPTB, which implies that the T4PP shows the highest resistance to the kinetics of charge transfer process associated with the corrosion reaction. For all the porphyrins studied, the inhibition efficiency increases with increasing concentration. In other words, the surface coverage of the inhibitors increases with increasing concentration. The trend of the values on *n* before and after the addition of the inhibitors also confirms the formation of protective films of the porphyrins on the N80 steel surface. This is because the values of *n* in the presence of inhibitors are generally higher than that of the uninhibited system. The lower *n* value for uninhibited solution (*n* = 0.616) can be attributed to surface inhomogeneity resulting from polishing scratches and/or corrosion products. The values of *n* lie between 0.722 and 0.864 for the inhibitor containing systems, which implies a reduction in surface inhomogeneity due to the adsorption of inhibitor molecules on the N80 steel surface [27,28,29,30].

The Bode plots also provide information about the capacitive and resistive behaviour of an electrochemical system. At intermediate frequencies (*S*), log |*Z*| *vs.* log *f* with slope values between −0.487 and −0.812 (close to −1), and the phase angle values for blank 3.5% NaCl (−31.4°), HPTB 200 ppm (−58.4°), T4PP 200 ppm (−57.3°), THP 200 ppm (−59.6°) and TPP 200 ppm (−60.5°) were observed (Table 2). These responses are characteristics of non-ideal capacitive behavior at intermediate frequencies. An ideal capacitive behavior would result in a slope of −1 and a phase angle of −90° [31,32]. Though the initial stage of immersion is usually marked with gradual approach of *S* and α to the ideal capacitive values, which may be associated with the reduction in the rate of metal dissolution with time. In this work, the faster attainment of steady state of *S* and α and their closer values to −1 and −90° respectively in the presence of inhibitors is another indication of the adsorption of the inhibitors on the steel surface [33].

**Table 1:** Electrochemical impedance parameters for N80 steel in 3.5% NaCl solution saturated with CO_2_ in without and with various concentrations of the studied porphyrins.

Aggressive Solutions	*R*_s_ (Ω·cm^2^)	*R*_ct_ (Ω·cm^2^)	*n*	*Y_o_* (Ω^−1^·s^n^·cm^-2^)	*L* (H·cm^2^)	*Chi*	η%
3.5% NaCl	10.0	121	0.616	129	33	0.0006	-
HPTB 50 ppm	1.4	600	0.728	95	20	0.0011	80
HPTB 100 ppm	1.1	622	0.818	99	28	0.0003	81
HPTB 200 ppm	1.8	818	0.858	102	22	0.0007	85
T4PP 50 ppm	1.9	612	0.722	116	22	0.0009	80
T4PP 100 ppm	1.3	743	0.827	78	19	0.0013	84
T4PP 200 ppm	1.7	1356	0.864	43	24	0.0007	91
THP 50 ppm	1.7	495	0.788	88	13	0.0008	76
THP 100 ppm	1.0	525	0.834	101	14	0.0006	77
THP 200 ppm	1.9	879	0.838	94	0	0.0012	86
TPP 50 ppm	1.8	429	0.816	102	14	0.0008	72
TPP 100 ppm	1.2	634	0.820	93	27	0.0008	81
TPP 200 ppm	1.4	986	0.825	99	21	0.0014	88

**Table 2:** The slopes of the Bode modulus plots at intermediate frequencies (*S*) and the maximum phase angles (α) of the Bode phase angle plots for N80 steel in 3.5% NaCl solution saturated with CO_2_ in absence and presence of porphyrins.

Aggressive Solution	−*S*	−α (°)
3.5% NaCl	0.487	31.4
HPTB (200 ppm)	0.808	58.4
T4PP (200 ppm)	0.745	57.3
THP (200 ppm)	0.723	59.6
TPP (200 ppm)	0.812	60.5

### 2.2. Polarization Measurements

**Figure 6:**
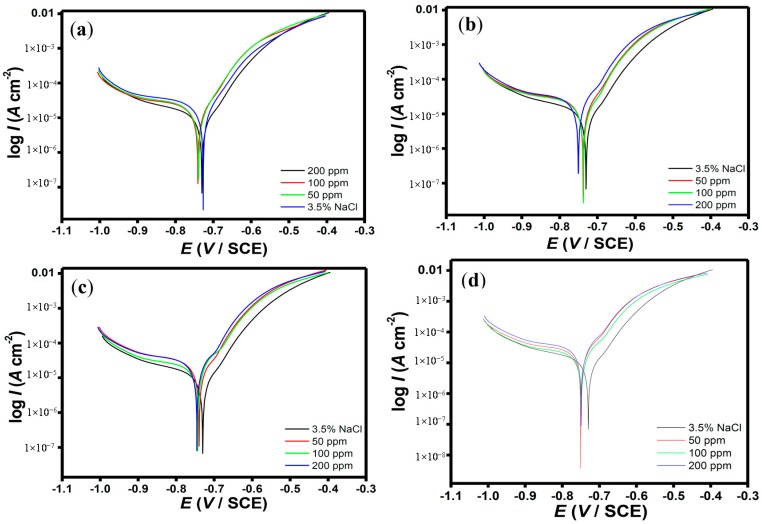
Potentiodynamic polarization curves of N80 steel in 3.5% NaCl saturated with CO_2_ without and with various concentrations of (**a**) HPTB; (**b**) T4PP; (**c**) THP and (**d**) TPP.

Potentiodynamic polarization curves for N80 steel at various concentrations of porphyrins in CO_2_-saturated 3.5% NaCl solution are shown in Figure 6a–d. Electrochemical kinetic parameters such as the corrosion current density (*I*_corr_), cathodic and anodic Tafel slopes (*b*_c_ and *b*_a_ respectively) were calculated by extrapolating the Tafel regions of the curves to the corrosion potential (*E*_corr_) and the inhibition efficiency, η% was calculated using the equation:

(3) $$\eta \%=\left(\frac{I_{\text{corr}}^{0}-I_{\text{corr}}^{\text{i}}}{I_{\text{corr}}^{0}}\right)\times 100$$

where *I*°_corr_ and *I*^i^_corr_ are the corrosion current density values without and with inhibitor respectively. The results are listed in Table 3.

It has been reported in the literature that a displacement in *E*_corr_ greater than 85 mV with respect to *E*_corr_ of the blank implies that the inhibitor can act as a cathodic or anodic type, while a displacement in *E*_corr_ less than 85 mV implies that the inhibitor is mixed type in action [34,35]. In the present study, the maximum shift in *E*_corr_ values is ±20 mV with relatively large change in the *b_c_* suggesting that porphyrins act as mixed type inhibitors but predominantly cathodic [36,37]. The value of *I*_corr_ decreases with increasing concentration of the inhibitors indicating that the inhibition efficiency increases with increase in concentration of the inhibitors.

**Table 3:** Tafel parameters for N80 steel in 3.5% NaCl solution saturated with CO_2_ in without and with various concentrations of the studied porphyrins.

Aggressive Solutions	*−E*_corr_ (mV *vs.* SCE)	*I*_corr_ (µA·cm^−2^)	*b_a_* (mV/dec)	*b_c_* (mV/dec)	η%
3.5% NaCl	730	60.77	69	214	-
HPTB 50 ppm	740	49.63	76	403	18
HPTB 100 ppm	742	31.19	90	583	49
HPTB 200 ppm	732	11.08	105	854	82
T4PP 50 ppm	738	44.68	67	285	26
T4PP 100 ppm	737	33.02	68	319	46
T4PP 200 ppm	750	9.13	69	282	85
THP 50 ppm	739	39.28	66	211	35
THP 100 ppm	745	28.77	65	203	53
THP 200 ppm	746	12.17	72	307	80
TPP 50 ppm	749	46.24	71	224	24
TPP 100 ppm	749	38.49	90	483	37
TPP 200 ppm	750	10.03	113	908	84

### 2.3. Scanning Electrochemical Microscopy (SECM)

**Figure 7:**
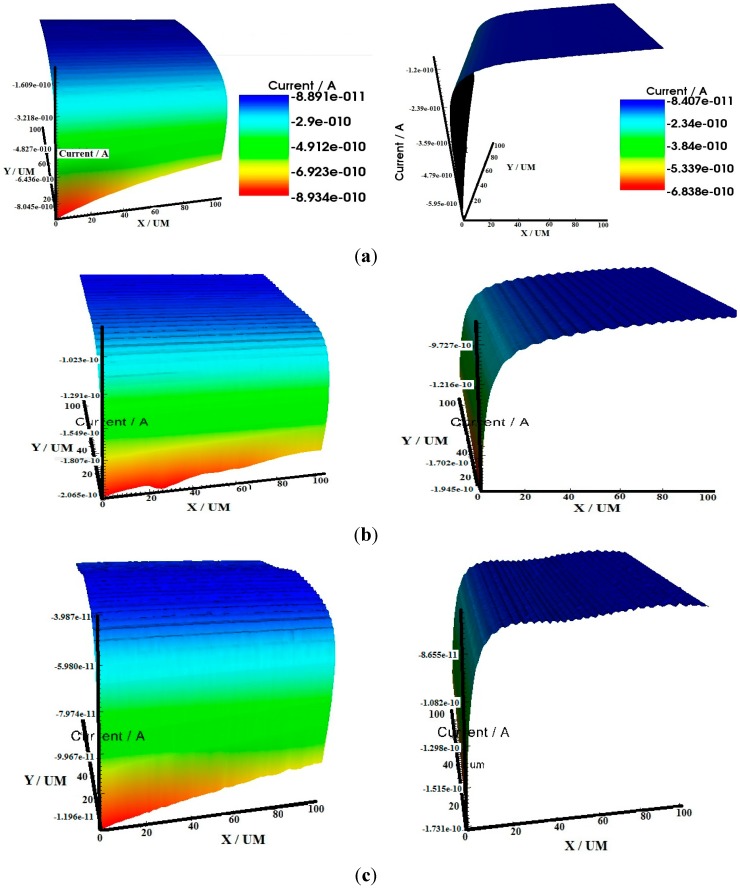

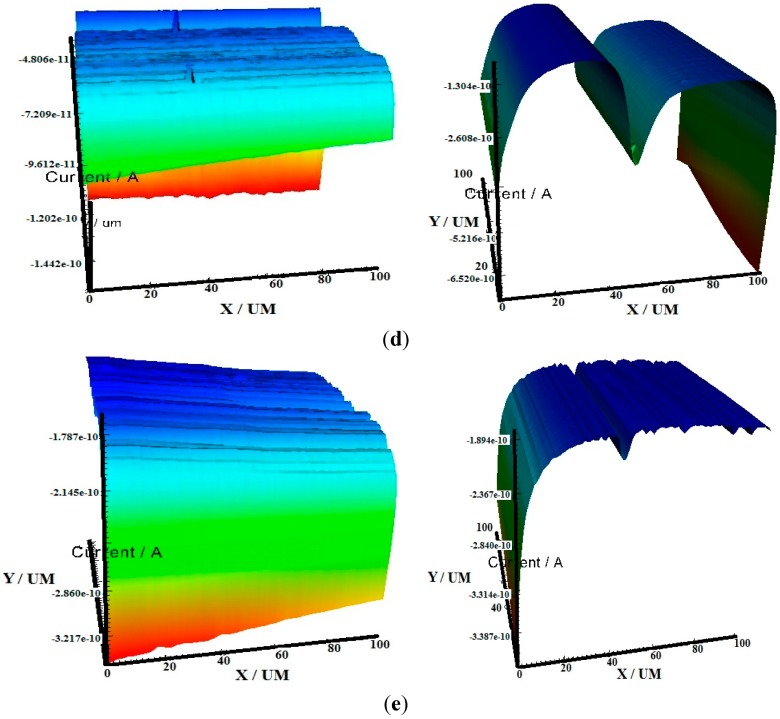
3D-SECM images (**left**: x-axis; **right**: y-axis) of N80 steel in 3.5% NaCl saturated with CO_2_ (**a**) without the inhibitors; and with 200 ppm (**b**) HPTB; (**c**) T4PP; (**d**) THP and (**e**) TPP.

The surface morphologies of the specimens visualized by SECM are shown in Figure 7. Prior to each SECM experiment, the distance between the tip and the sample was established by approach curve measurement performed above the insulating part of the sample at −0.70 V. The status of corroded sample was studied by monitoring the probe (tip potential: 0.5 V *vs.* Ag/AgCl, saturated KCl) and the substrate (tip potential: −0.7 V) in test solutions. Similar approach has been successfully applied and reported elsewhere [38].

As the tip approaches the surface of the N80 steel in the porphyrin containing aggressive solutions, the diffusion field surrounding the tip is hindered and the tip current decreases (Figure 7). This behaviour is typical of an insulating surface and confirmed that the steel surface is covered with protective film in the presence of the inhibitors [38]. In the absence of the porphyrin inhibitors, the tip current increases as the tip was made to approach the surface indicating that the N80 steel surface in the blank 3.5% NaCl solution is conductive and prone to a faster corrosion process.

### 2.4. Scanning Electron Microscopy (SEM)

**Figure 8:**
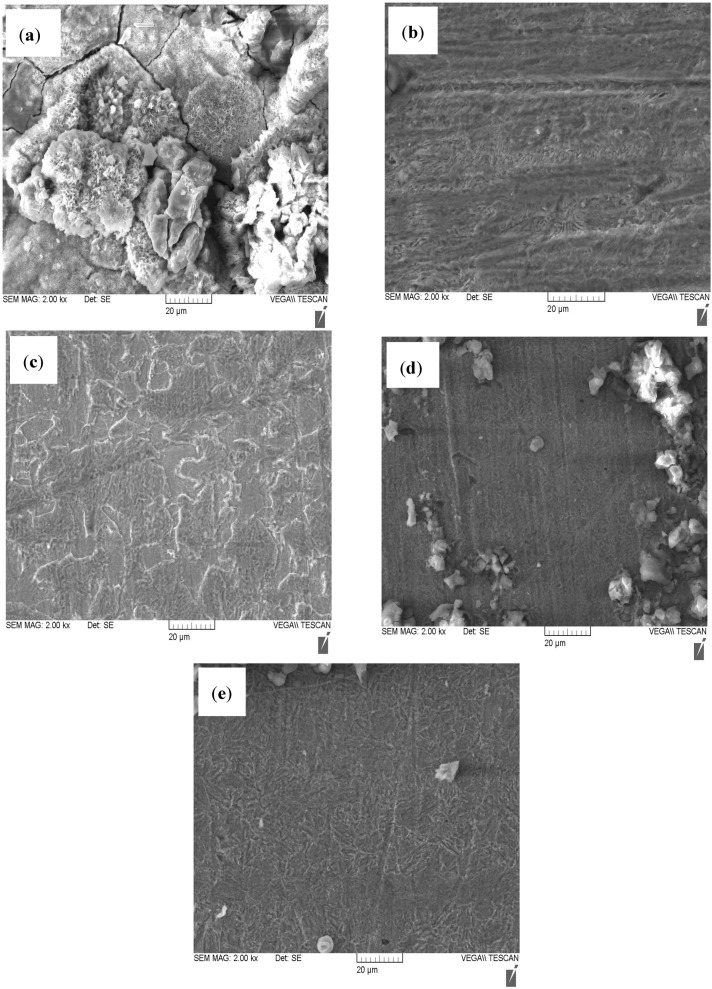
SEM images of N80 steel in 3.5% NaCl saturated with CO_2_ (**a**) without the inhibitors; and with 200 ppm (**b**) HPTB; (**c**) T4PP; (**d**) THP and (**e**) TPP.

Scanning electron microscopy images were taken to justify that the corrosion inhibition behaviour of the studied porphyrins is due to the formation of protective film on the steel surface. The SEM images of the N80 steel surfaces in 3.5% NaCl saturated with CO_2_ without and with 200 ppm of the inhibitors are shown in Figure 8. The morphology of the N80 steel surfaces in Figure 8a shows a corroded surface in the absence of inhibitors. There are pits and cracks on the N80 steel surface and the surface is intensely damaged. However, in the presence of the inhibitors, the surface corrosion of N80 steel is remarkably decreased showing less corroded and smoother surfaces as shown in Figure 8b–e. These results prove that the porphyrins used as corrosion inhibitors formed protective film on the N80 steel surface thereby protecting the surface from direct exposure to corrosive environments.

### 2.5. Quantum Chemical Studies

**Figure 9:**
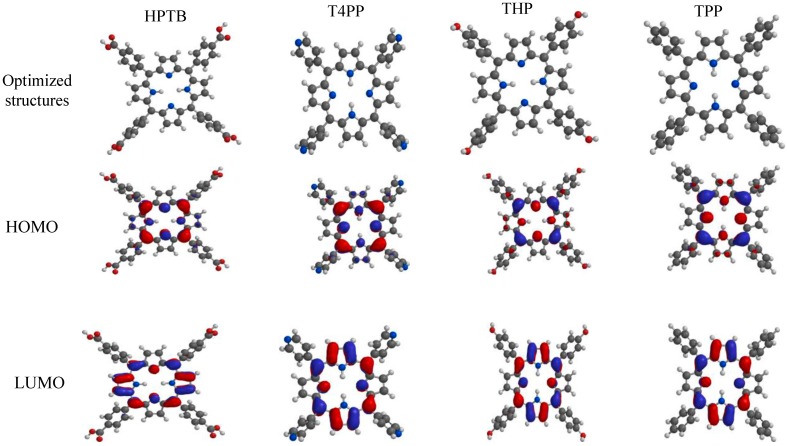
Optimized structures and the corresponding HOMO and LUMO electron density surfaces for the studied porphyrins.

The optimized geometries of the studied porphyrins are shown in Figure 9. All the quantum chemical data that are used for comparison with experimental results relate only to the lowest-energy conformer of each of the studied compounds. According to the frontier molecular orbital theory, chemical reactivity is strongly determined by the interaction of the highest occupied molecular orbital (HOMO) and the lowest unoccupied molecular orbital (LUMO) of the interacting species [39]. The HOMO and LUMO surfaces of the studied compounds are shown in Figure 9 alongside the optimized molecular structures. For all the studied porphyrins, the HOMO density is highest at C_5_, C_10_, C_15_, C_20_, N_22_, N_24_, N_21_ and N_23_ around the porphyrin ring. Also it is notable that there is significant HOMO density on the C_5a_, C_5f_, C_15a_ and C_20a_ atoms. This means that these atoms are the most likely atoms to donate electrons to the vacant *d* orbitals of the metal. The main contributing factor to the HOMO is the porphyrin ring because the substituent groups (in all the compounds) appear to have minimal or no HOMO density.

Other quantum chemical parameters were computed to have more insights into the reactivity and selectivity of the porphyrins. The frontier molecular orbital energies (*i.e*., E_HOMO_ and E_LUMO_) provide information on the reactivity of chemical species. The E_HOMO_ is often associated with the electron donating ability of a molecule [40,41,42] and a higher E_HOMO_ value indicates higher tendency of the molecule to donate electron(s) to the appropriate acceptor specie with low energy and empty/partially filled atomic/molecular orbitals. The results in Table 4 show that THP has the highest value of E_HOMO_ while T4PP has the lowest value. These results imply that THP has the highest tendency to donate electrons while T4PP has the lowest tendency to donate electrons. The high tendency of THP to donate electron may be related to the fact that it has the -OH group substituents, which is an electron donor to the attached ring. A -COOH group however is electron withdrawing, which explains why HPTB has lesser tendency to donate electrons than THP. Overall, adsorption of the porphyrins on metal surface is likely to follow the order of the electron donating ability as THP > TPP > HPTB > T4PP.

**Table 4:** Quantum chemical parameters for the studied porphyrins.

Inhibitors	E_HOMO_ (eV)	E_LUMO_ (eV)	ΔE (eV)	η (eV)	σ (eV)^−1^	ω (eV)	μ (Debye)	χ (eV)
HPTB	−5.31	−2.64	2.67	1.34	0.75	6.00	2.69	3.98
T4PP	−5.47	−2.74	2.73	1.37	0.73	6.17	0.11	4.11
THP	−4.74	−2.11	2.63	1.32	0.76	4.50	4.75	3.43
TPP	−4.90	−2.20	2.70	1.35	0.74	4.67	0.08	3.55

Molecules with large value of energy gap, ∆E (∆E = E_LUMO_ − E_HOMO_) are highly stable (*i.e*., they have low reactivity to chemical species) while molecules with small values of ∆E have high reactivity. A molecule with a small value of ∆E is easily polarized and can therefore be readily adsorbed on the metal surface, resulting in appreciably good inhibition efficiency. The data reported in Table 4 show that THP has the lowest ∆E followed by HPTB, which means that THP molecule could have better inhibition performance than the other corrosion inhibitors. Global electrophilicity (ω), electronegativity (χ), absolute hardness (η) and softness (σ) are other properties that are also often used to analyze the molecular reactivity and selectivity were calculated according to the equations [43]:

(4) $$\omega =\frac{\mu^{2}}{2\eta }$$

(5) $$\chi =\frac{1}{2}\left(I+A\right)$$

(6) $$\eta =\frac{1}{2}\left(I-A\right)$$

(7) $$\sigma =\frac{1}{\eta }$$

where *I* = −E_HOMO_ is the ionization potential and *A* = −E_LUMO_ is the electron affinity in accordance to the Koopman’s theorem [44]. 

The relationship between these quantum chemical quantities and corrosion inhibition is often based on the Lewis theory of acids and bases and the Pearson’s hard and soft acids and bases (HSAB) formalism [45]. A hard molecule has a large ∆E while a soft molecule has a small ∆E. Soft molecules therefore could easily offer electrons to an acceptor system which makes them more reactive than hard molecules. In this regard, adsorption could occur at the region of the molecule with the highest value of σ [46]. The values of σ reported in Table 4 show that the order of the σ values is THP > HPTB > TPP > T4PP. This trend is however, not entirely in agreement with the observed experimental inhibition efficiencies. The dipole moment (µ) is another index that is often used for the prediction of the trend of corrosion inhibition efficiencies. It is the measure of bond polarity and is related to the distribution of electrons in a molecule [47]. A survey of literature reveals that there are dissenting opinions on the correlation of dipole moments of inhibitor molecules with experimental inhibition efficiencies. One version of the opinions had suggested that inhibitors with high dipole moment tend to form strong dipole-dipole interactions with the metal, resulting in strong adsorption on the surface of the metal thereby leading to greater inhibition efficiency [48], while the other views had reported that low dipole moments will result in high inhibition efficiency due to favourable accumulation of the inhibitor molecules on the surface layer [34]. It has therefore been demonstrated that experimental inhibition efficiencies do not always correlate with dipole moments [49]. The results reported in Table 4 suggest that THP has the highest dipole moment (μ) magnitude, however, it has the lowest inhibition efficiency. Moreover, TPP and T4PP that have very low dipole moments have the highest inhibition efficiency values. These results suggest that for the studied porphyrin derivatives, the lower the dipole moment, the greater the inhibition efficiency, which is in line with the version of the opinions that suggests better adsorption of inhibitor molecules on the steel surface for a low dipole moment molecule [34].

The correlation of individual quantum chemical parameters with the inhibition efficiency of inhibitors is usually less informative because of the possible complexity of the adsorption process. It is therefore essential to combine several quantum chemical parameters to form a composite index that could be correlated to the experimental inhibition efficiency. A correlation between quantum chemical parameters and the observed inhibition efficiency is studied by means of quantitative structure activity relationship (QSAR) approach in which relevant mathematical equations are used to relate quantum chemical parameters to the observed inhibition efficiencies of inhibitors. The derived equations are used to predict the inhibition efficiency (η%) from the concentrations of the inhibitors and to provide theoretical explanations for the effects of different variables studied [39]. In the present work, the linear model proposed by Lukovits *et al* for the study of interaction of corrosion with metal surface in acidic solutions [50] was tested for the purpose of investigating the relationship between the reactivity parameters and inhibition efficiencies. The linear model equation has the form:

IE_theor_ = Ax_i_ C_i_ + B (8)

where A and B are the regression coefficients determined through regression analysis, x_i_ is a quantum chemical index characteristic of the molecule i, C_i_ is the experimental concentration of the inhibitor. The results of the QSAR analysis on the quantum chemical parameters obtained show that a combination of two quantum chemical parameters to form a composite index provides the best correlation with the experimental data. The best three equations obtained are:

η% = 441.39 − 172.707 × ∆N − 26.997 × ω, R^2^ = 0.965 and RMSE = 1.500 (9)

η% = −243.491 − 136.349 × E_HOMO_ + 149.978 × E_LUMO_, R^2^ = 0.991 and RMSE = 0.753 (10)

η% = 109.931 − 3.107 × ω − 2.153 × μ, R^2^ = 0.991 and RMSE = 0.783 (11)

The Equation (9) suggests that when ΔN and ω parameters are utilized, a lower ΔN and smaller ω result in greater inhibition efficiency. The Equation (10) suggests that a higher E_LUMO_ and lower E_HOMO_ result in greater inhibition efficiency. The Equation (11) suggests that high inhibition efficiencies could be obtained by lowering both ω and μ.

### 2.6. Monte Carlo Simulations

**Figure 10:**
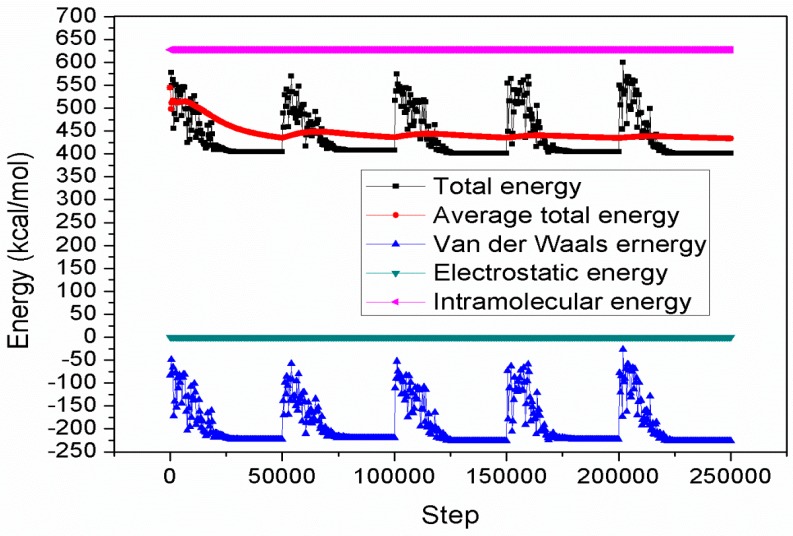
Total energy distribution for THP/Fe (110) surface.

**Figure 11:**
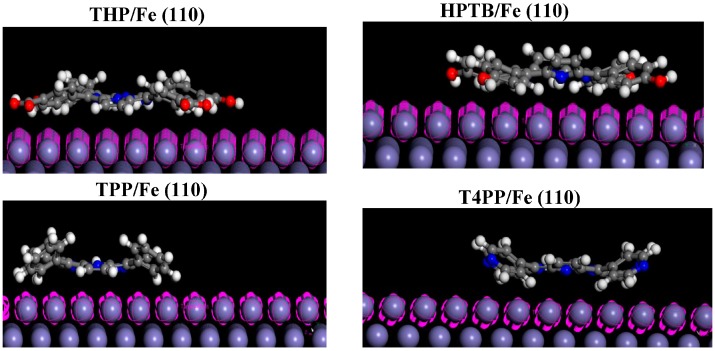
The most stable adsorption configuration of THP, HPTB, TPP and T4PP on Fe (110) surface using Monte Carlo simulations.

The values of the total energy, average total energy, van der Waals energy, electrostatic energy and intramolecular energy for the porphyrin/iron systems were calculated by optimizing the whole systems and a typical adsorption energy distribution is shown in Figure 10 for the THP/iron system. The most stable adsorption configurations of THP, HPTB, TPP and T4PP on Fe (110) surface using Monte Carlo simulations are shown in Figure 11. The corresponding values for the outputs and descriptors are listed in Table 5. The parameters include total energy of the substrate–adsorbate configuration, which is defined as the sum of the energies of the adsorbate components, the rigid adsorption energy, and the deformation energy. The substrate energy (*i.e*., Fe (110) surface) is taken as zero. Moreover, adsorption energy reports the energy released (or required) when the relaxed adsorbate component was adsorbed on the substrate. The adsorption energy is defined as the sum of the rigid adsorption energy and the deformation energy for the adsorbate component. The rigid adsorption energy reports the energy released (or required) when the unrelaxed adsorbate component (before the geometry optimization step) was adsorbed on the substrate. The deformation energy reports the energy released when the adsorbed adsorbate component was relaxed on the substrate surface. Finally, (dE_ad_/dNi) reports the energy of substrate–adsorbate configurations where one of the adsorbate components has been removed. 

It is quite clear from Table 5 that the adsorption energies of the studied inhibitors on iron surface increased in the order: THP < HPTB < TPP < T4PP. Highest negative adsorption energy indicates the system with the most stable and stronger adsorption [51]. This trend is in agreement with the values of global softness (σ) obtained using quantum chemical calculations.

**Table 5:** Outputs and descriptors calculated by the Monte Carlo simulation for adsorption of THP, HPTB, TPP and T4PP on Fe (110) surface (in kcal/mol).

Inhibitors	Total Energy	Adsorption Energy	Rigid Adsorption Energy	Deformation Energy	dE_ad_/dN_i_
THP	259.07	−368.11	−395.81	27.69	−368.11
HPTB	312.78	−301.66	−330.92	29.25	−301.66
TPP	395.21	−276.84	−295.42	18.57	−276.84
T4PP	393.89	−274.01	−294.15	20.14	−274.01

### 2.7. Mechanism of Corrosion Inhibition

The N80 steel inhibition process in the studied environment can be explained by the adsorption of the components of porphyrins on the metal surface. The adsorption of the active constituents of porphyrins on the N80 steel surface reduces the surface area that is available for the attack of the aggressive ion from the CO_2_ saturated 3.5% NaCl solution. It is impossible to consider a single adsorption mode between inhibitor and metal surface because of the complex nature of adsorption and inhibition of a given inhibitor [52,53,54,55]. The adsorption of main constituents of porphyrins can be attributed to the presence of N and O atoms, aromatic rings and conjugated π-electron systems. Therefore, the possible reaction centres are the unshared electron pair of hetero-atoms and π- electrons of the aromatic rings.

In aqueous solutions, the porphyrins exist either as neutral molecules or in the form of cations (protonated porphyrins). The neutral porphyrins may adsorb on the metal surface by displacing water molecules from the metal surface via the sharing of lone pair of electrons between the N and/or O atoms and iron. The porphyrin molecules can also be adsorbed on the metal surface on the basis of donor–acceptor interactions between the π-electrons and vacant d-orbitals of iron.

**Figure 12:**
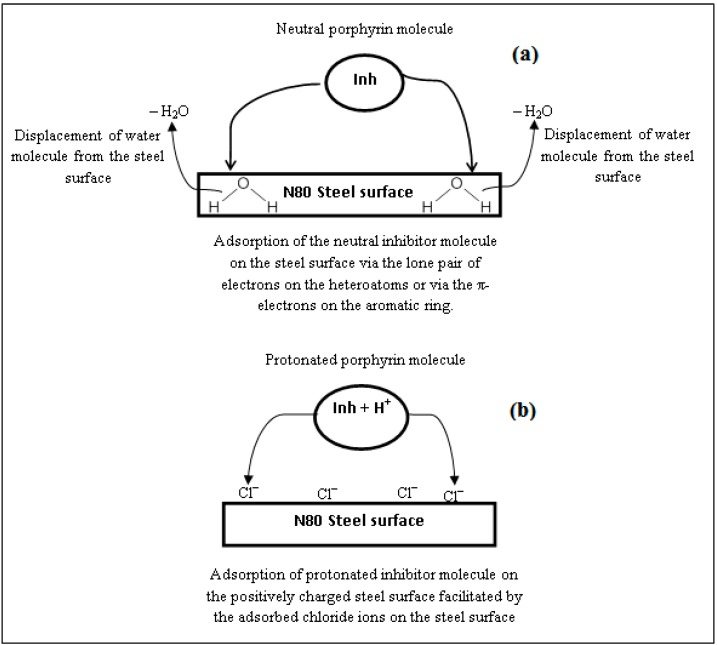
Possible mechanism of adsorption of the studied porphyrin molecules on the N80 steel surface.

The possible mechanism of adsorption of the studied porphyrin molecules on the steel surface is summarized in the schematic diagram as shown in Figure 12.

## 3. Experimental Section

### 3.1. Materials and Aggressive Solutions

Porphyrins were obtained commercially from Sigma Aldrich Chemicals (Johannesburg, South Africa) and used without further purification. Corrosion tests were performed on a N80 steel of the percentage composition (wt. %): C (0.31); Si (0.19); Mn (0.92); P (0.010); S (0.008); Cr (0.2) and Fe balance. The N80 steels were metallographically abraded according to ASTM A262 using fine grade emery papers of various grades (600 to 1200). The N80 steel coupons having the dimension 30 mm × 3 mm × 3 mm were used for the EIS and potentiodynamic polarization studies. The test solution of 3.5% NaCl was prepared by diluting the analytical grade NaCl with double distilled water.

### 3.2. Electrochemical Measurements

The electrochemical studies were conducted using conventional three-electrode cell assembly at room temperature. The freshly polished N80 steel was used as the working electrode, while platinum rod and standard calomel electrode (SCE, Metrohm, Johannesburg, South Africa) were used as auxiliary and reference electrodes respectively. All electrochemical measurements were carried out using Autolab Potentiostat/Galvanostat (Model PGSTAT302N) obtained from Autolab Instruments Inc. (Utrecht, The Netherlands). Prior to the electrochemical measurements, a stabilization period of 1 h was allowed to attain a stable value of corrosion potential (*E*_corr_).

Potentiodynamic polarization curves were obtained by sweeping the electrode potential from −300 to +300 mV *vs.* open circuit potential (OCP) at a scan rate of 1 mV·s^−1^. EIS measurements were carried out in a frequency range from 100 kHz to 0.00001 kHz with amplitude of 10 mV peak-to-peak, which had been adjudged reasonable to ensure linear impedance response that is independent of the perturbation amplitude [56]. The linear Tafel segments of anodic and cathodic curves were extrapolated to the *E*_corr_ to obtain corrosion current densities (*I*_corr_). The inhibition efficiency (η%) was evaluated from the measured *I*_corr_ values using the relationship:

(12) $$\eta \%=\frac{I_{\text{corr}}^{\text{0}}-I_{\text{corr}}^{\text{'}}}{I_{\text{corr}}^{\text{0}}}\times 100$$

where $I_{\text{corr}}^{0}$ and $I_{\text{corr}}^{'}$ are the corrosion currents in the absence and presence of inhibitor respectively. The charge transfer resistance values were obtained from the diameter of the semi circles of the Nyquist plots. The inhibition efficiency was also calculated from the charge transfer resistance values using the equation:

(13) $$\eta \%=\frac{R_{\text{ct}}^{\text{'}}-R_{\text{ct}}^{\text{0}}}{R_{\text{ct}}^{'}}\times 100$$

where, $R_{\text{ct}}^{'}$ and $R_{\text{ct}}^{0}$ are the charge transfer resistance in the presence and absence of inhibitor, respectively.

### 3.3. Scanning Electrochemical Microscopy (SECM)

SECM analysis was carried out in order to obtain spatial resolution of the electrode surface and to examine the electrochemical behaviour of the system at the inception of corrosion as well as to investigate possible defects on the surface. Since conventional electrochemical systems cannot provide such information, SECM has become one of the most powerful local techniques for corrosion research. It has a wide variety of operation modes, which contributes to its great versatility [57,58]. Freshly polished N80 steel coupon with the dimensions of 30 mm × 3 mm × 3 mm was used as the working electrode for the SECM studies. A 10 μm platinum tip was used as the probe while Ag/AgCl in saturated KCl and platinum rod were used as the reference and counter electrodes respectively. Line scan measurements were carried out by making a tip approach at ~10 μm from the surface of the N80 specimen. All measurements were carried out at the scan rate of 80 μm/step on a model CHI900C (CH Instruments, Inc., Austin, TX, USA) scanning electrochemical microscopy device.

### 3.4. Scanning Electron Microscopy (SEM)

Freshly polished N80 steel was immersed into the test solution (CO_2_ saturated 3.5% NaCl) in the absence and presence of the corrosion inhibitors. The N80 steel samples were retrieved from the aggressive solutions after 3 h of immersion, rinse with water and finally dried at an ambient temperature. Micrographs of freshly abraded and corroded N80 steel surfaces without and with inhibitors were taken using a SEM model VEGA II XMH instrument (TESCAN, Kohoutovice, Czech Republic).

### 3.5. Quantum Chemical Calculations

Gas phase geometry optimizations and vibrational frequency calculations were carried out on the studied porphyrins without symmetry constraint. The Becke three parameter hybrid functional together with the Lee-Yang-Parr correlation functional (B3LYP) was used for all the calculations [59]. The 6-31G (d,p) basis set was selected for the calculations. Optimized structures of the porphyrins were confirmed to correspond to true energy minima with the absence of imaginary frequency. All quantum chemical parameters were derived based on the electronic data of the optimized geometries. The calculated molecular parameters include energy of the highest occupied molecular orbital (E_HOMO_), energy of the lowest unoccupied molecular orbital (E_LUMO_), energy gap (∆E), dipole moment (μ), global softness (σ), global hardness (η), electrophilicity (ω), fraction of electrons transferred (∆N), electronegativity (χ), electron affinity (EA) and ionization potential (IE).

### 3.6. Monte Carlo Simulation Studies

Monte Carlo simulations using the adsorption locator code implemented in the Material Studio 6.0 software from Accelrys (San Diego, CA, USA) was adopted to compute the adsorption energy of the interaction between the inhibitor molecules and clean iron surface. The calculation was carried out using the Condensed-phase Optimized Molecular Potentials for Atomistic Simulation Studies (COMPASS) force field. Before simulations, the clean Fe (110) plane was first cleaved from Fe crystal, the surface was then optimized to the energy minimum. The Fe (110) plane was next enlarged to a (13 × 13) super-cell. After that, a vacuum slab with 5.0 nm thickness was built above the Fe (110) plane. Simulation annealing using Metropolis Monte Carlo method was used to sample possible configurations by carrying out Monte Carlo searches of the configuration space of the additives on the iron surface system as the temperature is gradually decreased.

## 4. Conclusions

Four porphyrin derivatives were investigated for their corrosion inhibition potential toward N80 steel corrosion in CO_2_-saturated 3.5% NaCl solution using EIS, polarization, SECM, SEM, quantum chemical calculations, QSAR and Monte Carlo simulation approaches. The follow conclusions can be drawn from the results.

(1) The studied porphyrins are good inhibitors for N80 steel corrosion in the studied media showing increasing η% with increase in concentration.
(2) The shift in *E*_corr_ (<85 mV) from polarization measurements indicated that the studied porphyrins acted as mixed type inhibitors.
(3) EIS data revealed that the porphyrins adsorbed on N80 steel surface and exhibit non-ideal capacitive behaviour.
(4) The SECM and SEM analyses showed that the porphyrins inhibit N80 steel corrosion by forming protective film on the steel surface.
(5) Quantum chemical parameters and QSAR analyses showed that the corrosion inhibition performances of the porphyrins could be related to their E_HOMO_, E_LUMO_, ω, and μ values.
(6) Monte Carlo simulation studies showed that THP has the highest adsorption energy, while T4PP has the least adsorption energy in agreement with the values of σ from quantum chemical calculations.
